# The *CLDN5* gene at the blood-brain barrier in health and disease

**DOI:** 10.1186/s12987-023-00424-5

**Published:** 2023-03-28

**Authors:** Yosuke Hashimoto, Chris Greene, Arnold Munnich, Matthew Campbell

**Affiliations:** 1 Trinity College Dublin, Smurfit Institute of Genetics, Dublin, D02 VF25 Ireland; 2 Institut Imagine, INSERM UMR1163, Université Paris Cité, Paris, F-75015 France; 3 Departments of Pediatric Neurology and Medical Genetics, Hospital Necker Enfants Malades, Université Paris Cité, Paris, F-75015 France

**Keywords:** Claudin-5, Blood–brain barrier, Tight junction, Psychiatric diseases, Vascular permeability

## Abstract

The *CLDN5* gene encodes claudin-5 (CLDN-5) that is expressed in endothelial cells and forms tight junctions which limit the passive diffusions of ions and solutes. The blood–brain barrier (BBB), composed of brain microvascular endothelial cells and associated pericytes and end-feet of astrocytes, is a physical and biological barrier to maintain the brain microenvironment. The expression of CLDN-5 is tightly regulated in the BBB by other junctional proteins in endothelial cells and by supports from pericytes and astrocytes. The most recent literature clearly shows a compromised BBB with a decline in CLDN-5 expression increasing the risks of developing neuropsychiatric disorders, epilepsy, brain calcification and dementia. The purpose of this review is to summarize the known diseases associated with CLDN-5 expression and function. In the first part of this review, we highlight the recent understanding of how other junctional proteins as well as pericytes and astrocytes maintain CLDN-5 expression in brain endothelial cells. We detail some drugs that can enhance these supports and are being developed or currently in use to treat diseases associated with CLDN-5 decline. We then summarise mutagenesis-based studies which have facilitated a better understanding of the physiological role of the CLDN-5 protein at the BBB and have demonstrated the functional consequences of a recently identified pathogenic CLDN-5 missense mutation from patients with alternating hemiplegia of childhood. This mutation is the first gain-of-function mutation identified in the *CLDN* gene family with all others representing loss-of-function mutations resulting in mis-localization of CLDN protein and/or attenuated barrier function. Finally, we summarize recent reports about the dosage-dependent effect of CLDN-5 expression on the development of neurological diseases in mice and discuss what cellular supports for CLDN-5 regulation are compromised in the BBB in human diseases.

## Introduction

**Fig. 1:**
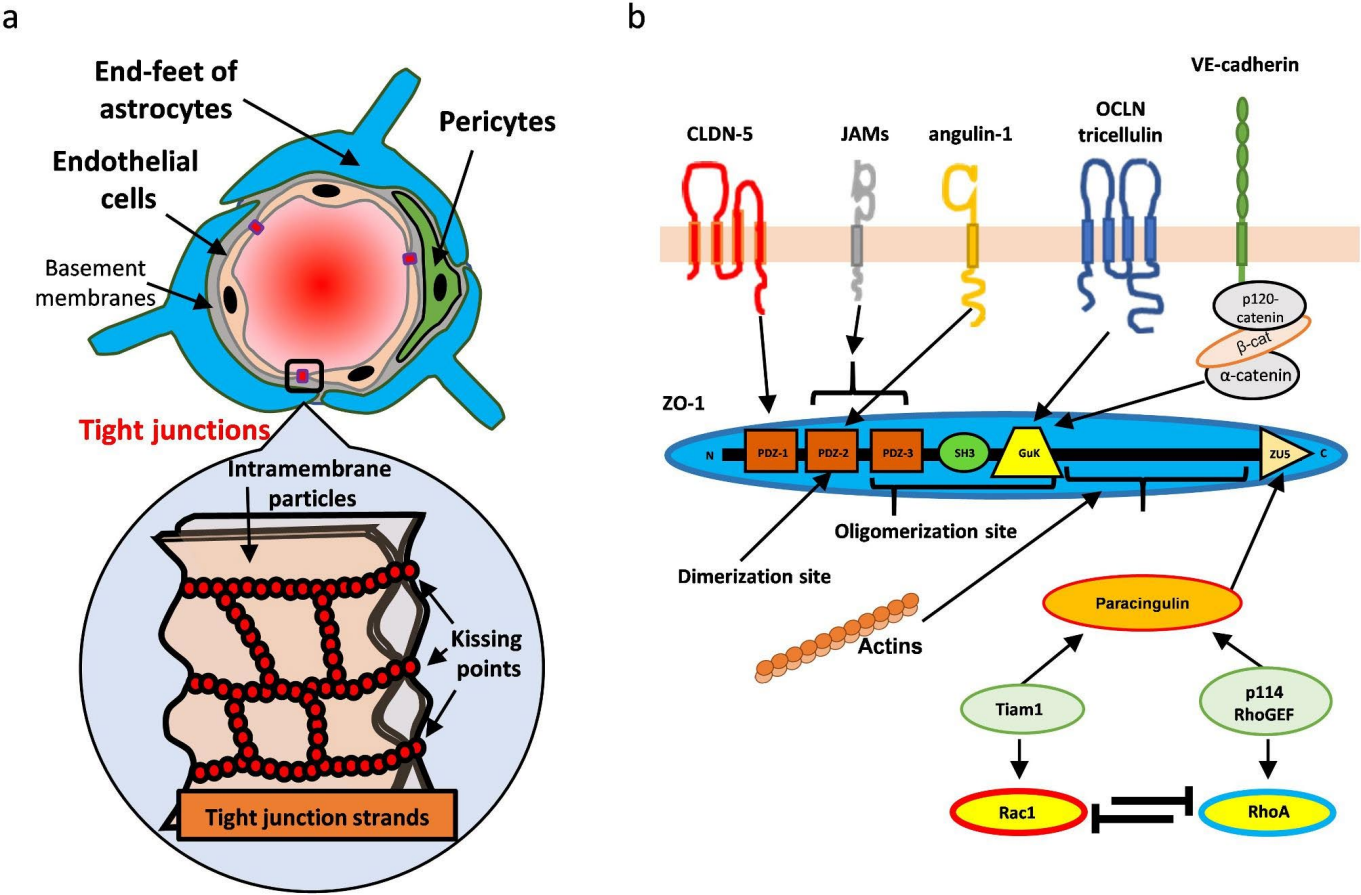
The schematic illustration of the BBB. (a) The BBB is composed of microvascular endothelial cells, pericytes and surrounded end-feet of astrocytes. One of the key features in brain microvascular endothelial cells is its well-developed tight junctions in their cellular clefts. Tight junctions can be observed as the mesh-like strands composed of intramembrane particles. At the membrane particles, the two plasma membranes are almost fused (also called kissing-points). (b) The junctional proteins in brain endothelial cells. VE-cadherin is a component of adherens junctions, but the subcellular localization of tight junctions and adherens junctions are almost same in brain endothelial cells. These proteins are interacted with ZO-1/-2 using different binding domain of ZO-1/-2. ZO-1 and -2 are also oligomerized by themselves. Paracingulin is a recruiter of guanidine exchange factors (GEFs) to junctional areas and GEFs are necessary to activate small GTPases Rac1 or RhoA. Rac1 strengthens the tight junctions while RhoA destabilizes the tight junctions and they inhibit each other

The brain capillaries are the major vasculature in the brain and represent approximately 85% of the vascular network [1]. Due to the density of the brain’s capillary network, it has been estimated that every neuron is nourished by its own capillary and their activity is correlated with regional cerebral blood flow (CBF) in an effort to gain adequate supply of oxygen and nutrients, known as a neurovascular coupling (NVC) or functional hyperemia. The blood–brain barrier (BBB), composed of brain microvascular endothelial cells (ECs) lining the wall of brain capillaries, is a physical barrier to separate the blood and central nervous system (CNS). The brain microvascular ECs exhibit some key structural and biological functions that peripheral microvascular ECs do not exhibit to maintain CNS homeostasis. Brain ECs have **(1)** abundant transporters/receptors to selectively recruit the required nutrients/molecules for CNS and efflux the metabolites/unnecessary molecules for CNS homeostasis, **(2)** no fenestration structures, **(3)** low pinocytosis activity, **(4)** high mitochondrial activity and **(5)** well-developed tight junctions (TJs) in cell-cell borders to prevent the random diffusion of molecules from blood. To acquire these properties, brain ECs receive many supports/signaling cues from associated pericytes, end-feet of astrocytes and basement membranes (Fig. 1a). The paracellular permeability of the BBB is maintained at a very low level; with only hydrophobic molecules (< 8 to 10 hydrogen bonds) less than approximately 400 Da able to pass [2].

TJs can be observed as mesh-like strands composed of polymerized intramembrane particles by using freeze-fracture replica electron microscopy [3]. At each intramembrane particle, adjacent plasma membranes are closely joined to each other to limit the paracellular route (known as “kissing points”) (Fig. 1a). Recent advanced super-resolution fluorescence microscopy can observe the TJ strands in live cells [4, 5]. The key structural component of the TJs is the claudin protein (CLDN) that polymerizes through cis- (in same cell) and trans- (between adjacent two cells) interactions. Twenty-seven *CLDN* genes are known to be present in mammals with 26 described in human. CLDNs are classified two categories based on amino acid sequence similarity: “classic” CLDNs (CLDN-1–10, -14, -15, -17, and -19) or “non-classic” CLDNs (CLDN-11, -12, -13, -16, -18, and -20–27) (CLDN-13 is absent in human). Most classic CLDNs can create TJ strands via homophilic trans-interaction [4, 6]. Some CLDNs clearly build TJs with a high barrier against ions and solutes as barrier-forming CLDNs (such as CLDN-1, -3, -5, -11, -18 and -19) or TJs with paracellular ion pores as channel-forming CLDNs (such as CLDN-2, -8 (with CLDN-4 as a recruiter), -10, -15, -16, -17 and -21) and the other CLDNs function as a barrier or channel depending on the expression levels of other CLDNs. They have different strength/preferences for cis- and trans-interactions and are expressed in a tissue-specific manner to create tissue-specific TJ strands/paracellular barriers. These TJ strands are highly dynamic, and a part of the strand is in a continuous state of annealing and breaking [5, 7]; in effect, solutes tend to pass through the transiently opened points in a step-by-step manner. CLDN-5 has been identified as one of the most important TJ proteins in the BBB. Indeed, *Cldn5*^*−/−*^ mice have increased BBB permeability against molecules less than 800 Da and die within 10 h of birth [8]. The expression of CLDN-12 and -25 in brain ECs has also been confirmed but studies using single cell or nucleus RNA sequencing clearly showed that the dominant CLDN in brain ECs appears to be CLDN-5 in both human and mouse [9, 10]. CLDN-5 expression is highly regulated by endothelial specific transcriptional regulators (Table 1), but CLDN-12 and -25 are not; these CLDNs are expressed in many tissues and cell types with low to intermediate expression level except for CLDN-25 in oligodendrocytes [9]. Added to this, *Cldn12*^*lacZ/lacZ*^ mice do not show BBB impairment [11] and the biological role of CLDN-25 in the BBB or even in other cells remains unknown.

In brain ECs, the TJ strands composed of CLDN-5 also contain occludin, tricellulin, junctional adhesion molecules (JAMs) and angulin-1 (also known as lipolysis-stimulating receptor; LSR) (Fig. 1b) [9]. These membrane proteins are connected to the actin cytoskeleton via binding to different domains of zonula occludens-1 (ZO-1) [12–16]. Adherens junctions (AJs) created by VE-cadherin recruit ZO-1 or ZO-2 at nascent cell-cell contacts and then ZO-1 and ZO-2 dimerization/oligomerization form scaffolds to mature the TJs [17]. Cells lacking both ZO-1 and -2 fail to develop TJ strands [6]. JAM-A forms close membrane apposition after the AJs are developed and is required to initiate TJ maturation by the accumulation of CLDNs to ZOs [6, 18]. Therefore, VE-cadherin and JAM-A are necessary for the barrier maturation process and act as an upstream regulator of CLDN-5 localization into the TJs. Occludin preferentially localizes to the branching site of the TJ strands [5] and is necessary to increase the complexity of the CLDN-based mesh-like strands [19]. The presence of tricellulin and LSR is necessary to build the CLDN-based strands at tricellular contacts where the corners of three cells meet [20]. Paracingulin and cingulin are recruited to ZO-1 with guanine nucleotide exchange factors (GEFs), such as p114RhoGEF and Tiam1 [21]. GEFs can activate small GTPases; Tiam1 can activate Rac1 to stabilize the TJs by forming cortical actin belts while p114RhoGEF can activate RhoA to cause the opposite reaction. Rac1 is important for barrier maturation while RhoA is important for angiogenesis and pathological events. The expression and barrier function of CLDN-5 is maintained by these junctional proteins with pericytes and the end-feet of astrocytes further supporting junctional stabilization by secreting ligands for G-protein coupled receptors (GPCRs) (Table 2) and growth factors. Some of these regulatory systems have been shown to be disturbed in CNS diseases [1, 22, 23], resulting in reduced CLDN-5 expression to initiate or worsen the pathological conditions.

**Table 1:** Reported repressors or enhancers of *CLDN5* promoter

Transcriptional factors	Ref
**Enhancers**	
E26 transformation specific (ETS)-1	[31, 93]
ETS-related gene (ERG)	[29]
Sex-determining region Y-box 18 (SOX-18)	[28]
Kruppel-like factor 4	[30]
CCAAT/enhancer-binding protein-α (C/EBP-α)	[52]
Glucocorticoid receptor	[38]
Estrogen receptor β/Sp1	[39, 40]
Vitamin D response element	[41]
**Repressors**	
NF-κB subunit p65 (RelA)	[46]
SMAD2/SMAD3/SMAD4/β-catenin	[90]
Runt-related transcription factors 1 (RUNX1)	[47]
ZONAB	[121]
β-catenin/FoxO1/Tcf4/Suz12/Ezh2/Eed	[57]

Eed, embryonic ectoderm development; Ezh2, enhancer of zeste homolog 2; FoxO1, forkhead box protein O1; Suz12, suppressor of zeste 12; Tcf4, transcription factor 4; ZONAB, ZO-1-associated nucleic acid binding protein

**Table 2:** Major GPCRs in brain ECs

Gene	Ligands	Gα subunits	Notes	Ref
Sphingosine 1-phospate receptors (S1PR)				
*S1PR1*	Sphingosine 1–phosphate	i/o	Endothelial specific *S1pr1*^*−/−*^ mice have loosened BBB with altered CLDN-5 localization	[65]
*S1PR2*		12/13	Its expression was induced by hypoxic stress in the ECs for promoting angiogenesis.	[112]
*S1PR4*		i/o, 12/13	Selective S1PR4 antagonist (CYM50358) could loosen the BBB in mice	[66]
Frizzled receptors (FZDs)				
*GPR124*	Wnt7a/b	-	There is an atypical Wnt7a/b-specific co-receptor complex with Reck/GPR124/FZDs in brain ECs to stabilize the TJs	[73]
Lysophosphatidic acid (LPA) receptors				
*LPAR4*	18:1 LPA	s, i/o, q/11, 12/13	*Lpar4*^*−/−*^ mice show attenuated LPA-induced hypertensive response in a Gα_12/13_ dependent manner.	[111]
*LPAR6*	2-acyl-LPA	12/13	*Lpar6*^*−/−*^ mice have a decreased vascular density and branching and show attenuated LPA-induced hypertensive response	[111]
Receptors for metabolic waste products				
*GPR4*	CO_2_/H^+^	q/11, s, i/o, 12/13	Gα_q/11_ is used for CO_2_-mediated vasodilation. Gα_s_ activation is also reported.	[131]
*HCAR1*	Lactate	i/o	It is expressed in both luminal and abluminal side.	[71]
Receptors for prostaglandins (PGs)				
*PTGER1*	PGE_2_	q/11	Responsible PGE_2_ receptor for vasodilation via PGE_2_ released from activated CNS cells	[116]
*PTGER2, 4*	PGE_2_	s	Its expression in brain ECs was induced by ischemia-reperfusion injury.	[107]
Other important GPCRs in the BBB				
*CALCRL*	Adrenomedulin	s	Fluid shear stress-induced Piezo-1 activation induces adrenomedullin.	[104]
*P2RY1, 2*	ATP	q/11	Fluid shear stress-induced Piezo-1 activation induces ATP. Activated CNS cells also release ATP.	[106]
*ADORA2A*	Adenosine	s	Adenosine mainly arises from the hydrolysis of released ATP.	[105]
*SMO*	SHH bound PYCH-1	i/o	Active Gα_i_ is necessary for efficient activation of Gli transcriptional factors.	[76]
*F2R*	Itself	12/13, q/11	A lymphocyte-released serine protease or thrombin can activate it	[113]
*HRH1*	Histamine	q/11	Endothelial dysfunction induced by histamine is dependent on Gα_q/11_ and RhoA	[132]
*BDKRB2*	Bradykinin	q/11	RMP-7 bradykinin analog is a representative BBB opener	[133]
*GPR55*	Lysophosphatidylinositol	q/11, 12/13	GPR55 is also activated by endocannabinoids and synthetic cannabinoid ligand	[134]

PTCH-1, patched-1; SHH, Sonic hedgehog;

## The regulatory mechanisms of CLDN-5 by junctional proteins and associated signaling in the brain ECs

### *CLDN5*, the down-stream gene of junctional proteins

**Fig. 2:**
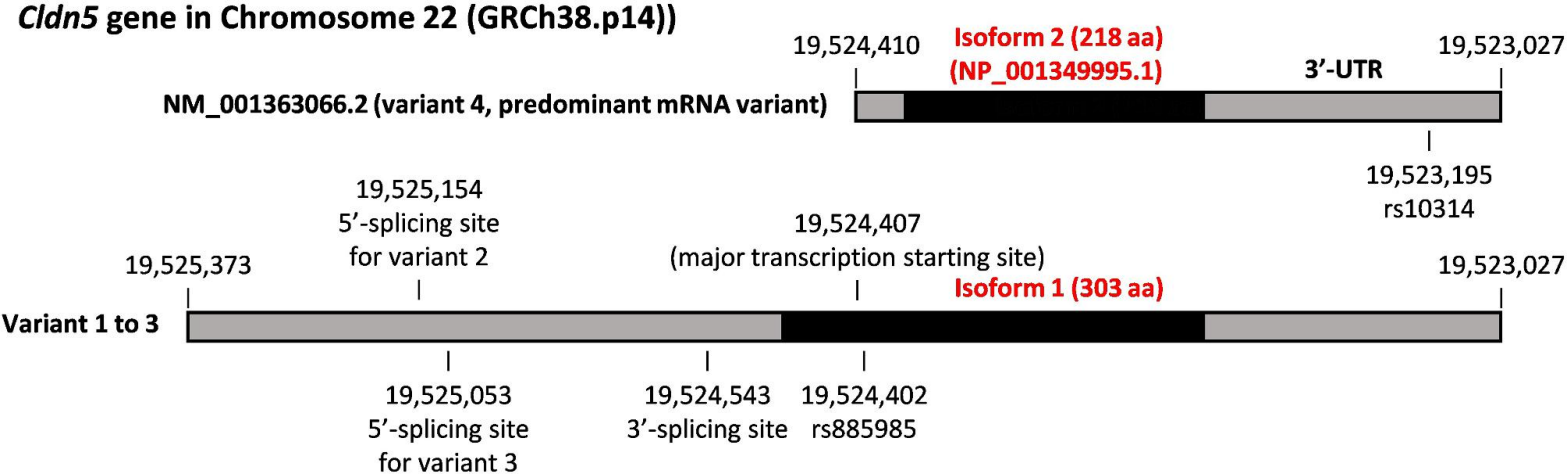
The basic information of *CLDN5* gene The key characteristics of transcriptional variants of *CLDN5*. The open-reading frames are highlighted by black bars

*CLDN5* was originally identified as one of the deleted genes in 22q11 deletion syndrome (22q11DS), also known as DiGeorge syndrome or Velocardiofacial syndrome, and was named *TMVCF* (transmembrane protein deleted in velocardiofacial syndrome) [24]. In humans, 4 mRNA transcripts are registered in the NCBI database; transcript variant 4 encodes a shorter CLDN-5 protein (isoform 2) and the other transcriptional variants encode an N-terminally extended longer CLDN-5 protein (isoform 1) (Fig. 2). The major transcription initiation site is located between the start codon of isoforms 1 and 2 and variant 4 is the predominant mRNA variant. Although a prediction tool suggests that the start codon of the longer isoform encoding transcriptional variants is functional [25], the longer isoform of CLDN-5 has not been detected by Western blotting. In addition, the longer CLDN-5 isoform expressed by the CMV promoter and an artificial Kozak sequence showed minimal localization onto the cell surface [26]. One single nucleotide polymorphism (SNP) whose allele frequency is almost 50% is located 3 nucleotides upstream from the major transcription initiation site. This SNP, *rs885985*, creates a stop codon and a short open reading frame in transcriptional variants 1 to 3, but the effect of this short open reading frame on the expression of the shorter CLDN-5 isoform is still unclear. Rodents appear to have only one mRNA transcript encoding the shorter isoform of CLDN-5.

Many repressors and enhancers of the *CLDN5* promoter for both longer and shorter transcripts have been identified using luciferase-based promoter assays or chromatin immunoprecipitation assays (Table 1) [27, 28]. Key transcriptional factors for ECs such as E-26 transformation specific (ETS) related gene (ERG), ETS-1, sex-determining region Y-box 18 (SOX-18), and Krüppel-Like Factor 4 (KLF-4), bind to the *CLDN5* promoter as an enhancer [28–31]. ERG, ETS-1 and KLF-4, but not SOX-18, also bind to the VE-cadherin (*CDH5*) promoter to enhance transcriptional activity [32–34]. KLF-4, SOX-18 and ETS-1 are up-regulated by shear stress [35–37], but aged ECs become less responsive to shear stress. Some steroid hormone receptors, including estrogen receptors, also function as a *CLDN5* enhancer [38–41]. Likely due to the effect of estrogen receptors, it has been shown that women have a stronger resistance against age-related increases in BBB permeability than men until the late life-stage [42]. After the end of estrogen production, the BBB in the occipital cortex, where the estrogen-producing neurons are highly enriched [43], becomes more vulnerable in women [42]. *CLDN5* expression is also regulated by circadian rhythms and clock transcription factor brain and muscle aryl-hydrocarbon receptor nuclear translocator like protein 1 (BMAL1); the expression of *Cldn5* is higher in the morning and lower in the evening, with rhythmic expression lost in endothelial specific *Bmal1*^*−/−*^ mice [44, 45]. Human and non-human primates also showed higher retinal vascular permeabilities in the evening compared with the morning [44].

Inflammatory mediators can function as *CLDN5* transcriptional suppressors. ERG, which is the most abundantly expressed ETS family member in ECs, is down-regulated by inflammatory cytokines [29] and there are also some nuclear factor κB (NF-κB) binding sites that can repress the *CLDN5* promoter [46]. Runt-related transcription factors 1 (RUNX1), which is a *CLDN5* repressor [47], is upregulated by tumor necrosis factor-α via c-Jun N-terminal kinases (JNK) pathway, not NF-κB pathway [48]. These mediators also compromise the trans-interaction of VE-cadherin and JAM-A; however, these inflammatory mediators up-regulate JAM-A expression because JAM-A has a non-junctional, proinflammatory function that enhances neutrophil extravasation by interacting with integrins on neutrophils [49].

Almost all CLDN family members (including CLDN-5) have a YV-motif at their C-terminus that binds to the N-terminal PDZ domain (PDZ1) of ZO-1 and -2 (Fig. 1b). The PDZ1-YV motif interaction is not necessary for junctional localization of CLDNs, but is necessary for its proper junctional stabilization by ZOs [4, 5, 16, 50]. The phosphorylation of T207 by Rho-associated kinases (ROCKs) disturbs its interaction and causes attenuated junctional localization of CLDN-5 [51]. CLDN-5 is incorporated into TJs after the trans-interaction of VE-cadherin and JAM-A are established via ZO-1. VE-cadherin, JAM-A and ZO-1 can control the junctional localization of CLDN-5 via Rac1/RhoA balance, and, moreover, they also control transcriptional activity of the *CLDN5* gene [21, 52, 53].

### VE-cadherin-mediated *CLDN5* regulation and PI3K/Akt signaling from pericytes and astrocytes

**Fig. 3:**
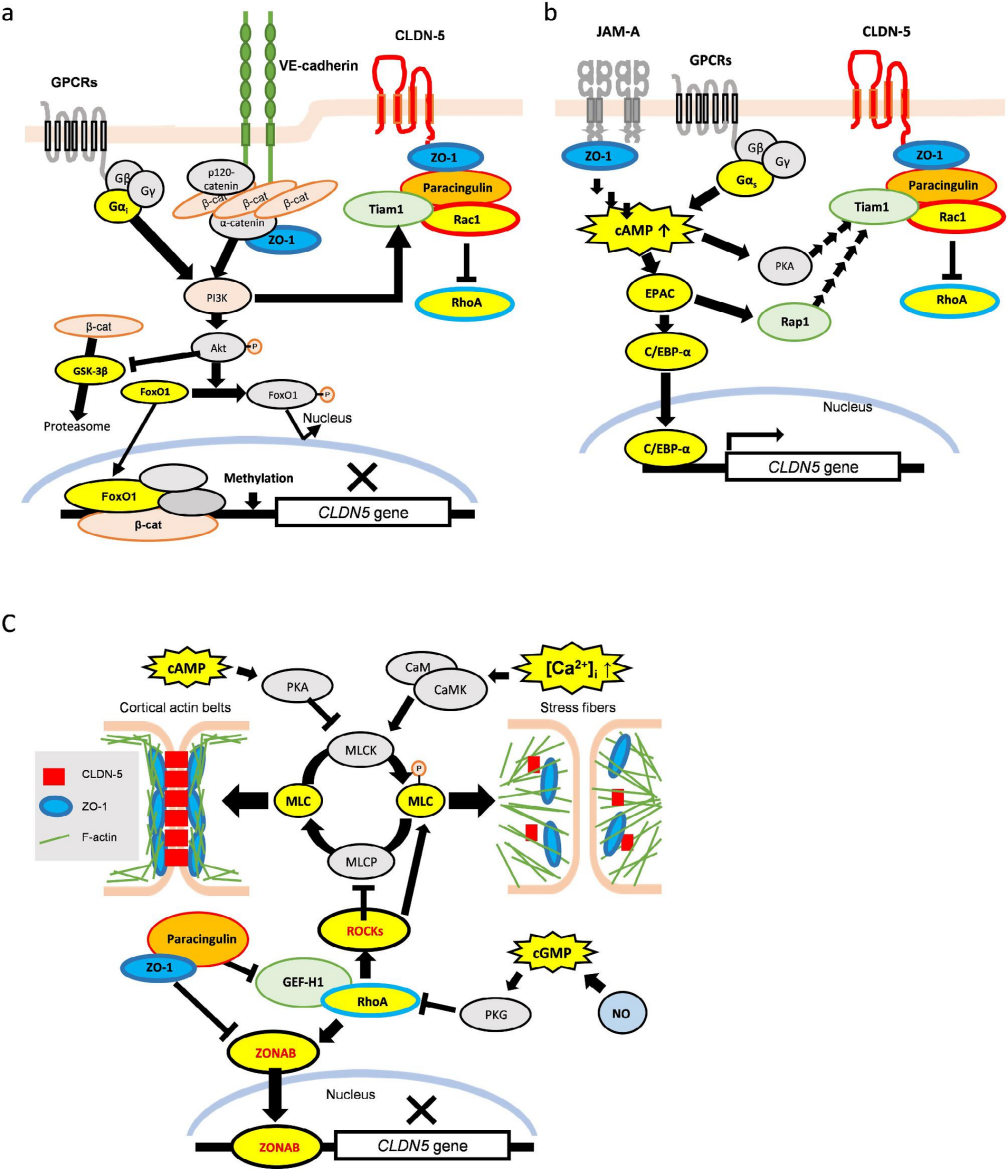
The TJ proteins mediated *CLDN5* regulation in the brain endothelial cells (a) VE-cadherin-mediated *CLDN5* regulation via PI3K/Akt signaling. The trans-interaction of VE-cadherin activates PI3K/Akt signaling and leads Rac1-mediated junctional stabilization and inhibits β-catenin/FoxO1-mediated *CLDN5* suppression. (b) JAM-A-mediated *CLDN5* regulation via cAMP signaling. The trans-interactions of JAM-A enhances cAMP level and leads protein kinase A (PKA)-dependent barrier stabilization via Rac1 activation and PKA-independent *CLDN5* up-regulation via C-EBP-α. (c) ZO-1-mediated *CLDN5* regulation by preventing non-junctional RhoA activation. Well-stabilized ZO-1 prevents the translocalization of ZO-1-associated nucleic acid binding protein (ZONAB) to the nucleus by direct interaction and by inhibiting non-junctional GEF-H1 activation via paracingulin. RhoA activation induces cell contraction by accumulated phosphorylated myosin light chain (MLC). Nitric oxidase (NO) and cAMP signaling inhibits cell contraction, but calcium signaling promotes it. CaM, calmodulin; CaMK, calmodulin kinase; C/EBP-α, CCAAT/enhancer-binding protein-α; eNOS, endothelial nitric oxide synthase; EPAC, exchange proteins directly activated by cAMP; GSK-3β, glycogen synthase kinase-3β; MLC, myosin light chain; MLCK, myosin light chain kinase; MLCP, myosin light chain phosphatase; PI3K, phosphoinositide 3-kinases; PKA, protein kinase A; PKG, protein kinase G

The trans-interactions of VE-cadherin activates phosphoinositide 3-kinase (PI3K) that can enhance the junctional localization of Tiam1 to stabilize the TJs via Rac1 activation (Fig. 3a). PI3K also activates Akt (protein kinase B) which can inhibit glycogen synthase kinase-3β (GSK-3β) activity, resulting in increased intracellular β-catenin levels. The wingless/int-1 (Wnt) receptor complex composed of low-density lipoprotein receptor-related protein (LPR)-5/6 and Frizzled receptors also inhibits GSK-3β via activation of Disheveled and induces/suppresses gene expression by transcriptional factor complexes with β-catenin. VE-cadherin mediated accumulation of β-catenin at junctions also stabilizes CLDN-5 expression by extending its half-life [54]. The half-life of CLDN-5 in cultured ECs (primary human brain microvascular ECs, human umbilical vein ECs or bovine retinal ECs) differ depending on the experimental conditions (varying between 1 and 14 h) probably due to differences in supporting structures [54–56]. However, when not sequestered by VE-cadherin at junctions, cytosolically accumulated β-catenin enters the nucleus and interacts with FoxO1 and/or other transcriptional factors, although Akt can inhibit the nuclear localization of FoxO1 by phosphorylation [53]. The transcriptional complex of β-catenin and FoxO1 epigenetically suppresses *CLDN5* transcription by promoter methylation and promotes angiogenic responses [57]. PI3K/Akt signaling functions like Wnt/β-catenin signaling and does not activate Rac1 in angiogenic and proliferative states [58], but PI3K/Akt and Wnt/β-catenin signaling induces Rac1 activation and extends the CLDN-5 half-life after the AJs are established.

The coverage of pericytes is also very important to mature the CLDN-5-based barrier in ECs by activation of PI3K/Akt signaling. ECs secrete PDGF-β (platelet-derived growth factor-β) to recruit pericytes to the vasculature via PDGFR-β (PDGF receptor-β) on pericytes [59]. The interaction of EphrinB2 in ECs and EphB4 on pericytes also recruits pericytes to the ECs especially after an acute inflammatory response [60]. N-cadherin forms junctions between ECs and pericytes and then activates PI3K/Akt signaling [61]. Angiopoietin-1 and sphingosine-1-phosphate (S1P), a sphingolipid metabolite, are released by pericytes to activate Tie2 and S1P receptor 1 (S1PR-1), respectively, in brain ECs to mature the TJs [62–64]. Endothelial specific *S1pr1*^*−/−*^ mice have increased BBB permeability with altered CLDN-5 localization [65]. S1PR-1 and -4 are GPCRs mainly coupled with Gα_i/o_ subunits and function as a barrier stabilizer for ECs through PI3K/Akt signaling [66]. Pericytes at the BBB are subdivided into mesh pericytes and thin-stranded pericytes by their morphologies [67]. The mesh pericytes cover larger areas of ECs than thin-stranded pericytes, indicating that the BBB composed of mesh pericytes may be tighter. Their contractile ability is relatively unknown, but ensheathing pericytes that are present in the precapillary arterial ECs have contractile abilities like smooth muscle cells and control CBF. CD146 (or melanoma cell adhesion molecule; MCAM) is a co-receptor of PDGFR-β and mediates pericyte recruitment to ECs where it displays a dynamic pattern of expression. Initially expressed by immature ECs, its expression pattern changes to pericyte-specific with increasing coverage of pericytes to ECs. Endothelial-specific deletion of CD146 resulted in BBB breakdown and reduced brain EC CLDN-5 expression, while pericyte-specific deletion resulted in reduced pericyte coverage and BBB breakdown [68].

Astrocyte end-feet are also an important regulator of PI3K/Akt signaling in brain ECs. Growth/differentiation factor-15 (GDF-15), secreted by astrocytes, enhances perivascular interaction between astrocytic end-feet and ECs but is also responsible for enhancing CLDN-5 expression in brain ECs cultured in astrocyte conditioned media via PI3K/Akt signaling [69, 70]. Some ligands released from astrocytes also activate Gα_i/o_ subunits via GPCRs in ECs. A glucose metabolite, lactate, is secreted from astrocytes and its receptor, hydroxycarboxylic acid receptor 1, is expressed in both luminal and abluminal membrane of brain ECs [71]. Among Wnt ligands, Wnt7a/b secreted by astrocytes are important for barrier maturation in brain ECs that have an atypical Wnt7a/b-specific co-receptor complex with Reck/GPR124/Frizzled/LRP-5/6 [9, 72, 73]. Sonic hedgehog secreted by astrocytes inactivates its receptor Patched-1 (Ptch-1), allowing activation of Smoothened (Smo) in brain ECs [74–76]. The activated Smo activates associated Gli-1 transcription factor that can activate SOX-18 to induce *CLDN5* expression [75]. The morphologies/functions of astrocytes are different in white matter (fibrous astrocytes) and gray matter (protoplasmic astrocytes) [77, 78], but their difference regarding barrier maturation in brain ECs is still not clear.

Many inflammatory factors change the phosphorylation status of VE-cadherin and its adaptor proteins, p120-cateninin and β-catenin, leading to the internalization of VE-cadherin through the various kinases [79]. Vascular endothelial growth factor A (VEGFA)-mediated phosphorylation of Y949 on the VEGF receptor 2 (VEGFR2) induces the phosphorylation of Y685 in VE-cadherin. In turn, leukocytes induces SH2 domain-containing protein tyrosine phosphatase-2 (SHP-2)-mediated dephosphorylation of Y731 on VE-cadherin [80, 81]. The phosphorylation of Y685 or dephosphorylation of Y731 of VE-cadherin induces VE-cadherin internalization. Vascular endothelial receptor-type protein tyrosine phosphatases (VE-PTPs), which is also epigenetically suppressed by the β-catenin/FoxO1 transcriptional repressor, induces dephosphorylation of Y685 on VE-cadherin [57, 81]. The activity of matrix metalloprotease-2/-9 (MMP-2/-9), whose major sources is infiltrated neutrophils, is also controlled by pro-inflammatory cytokines and chemokines; CLDN-5 is not a direct substrate of MMP-2/9 [82], but VE-cadherin is a direct substrate of MMP-9 [83]. In addition, a disintegrin and metalloproteinase (ADAM)-10 cleave VE-cadherin [84]. ADAM-10 also sheds PDGFR-β from pericytes [85]. The increased intracellular Ca^2+^ level is necessary to activate ADAM-10 [86]. The degradation of extracellular matrices also affects the junctional localization of VE-cadherin in ECs and impairs the adhesion by integrin-β1 that induces PI3K/Akt signaling [87, 88].

After the trans-interaction of VE-cadherin is established, transforming growth factor-β (TGF-β) signaling is amplified because VE-cadherin helps the assembly of the receptor complexes for TGF-β [89]. The receptor complex of type I TGF-β receptor (activin receptor-like kinase-5, ALK-5) and type II TGF-β receptor (TGFBR-2) can activate Smad2/3, which interact with free β-catenin and transcriptionally suppress *CLDN5* expression, upon TGF-β stimulation [90]. ALK-5-mediated signals induce cell migration and trans-differentiation into fibroblasts or pericytes (endothelial-to-mesenchymal transition, EndMT) [91]. An ALK-5 inhibitor is necessary to differentiate ECs from pluripotent stem cells [92, 93] while the ALK-5/TGFBR-2 complex with additional type I TGF-β receptor, ALK-1, can activate Smad1/5 and inhibit Smad2/3 signaling upon TGF-β or bone morphogenetic protein-9 stimulation. ALK-1-mediated signaling prevents phosphorylation of Y949 on the VEGFR2 to mature the barrier [94]. The expression level of ALK-1 is much higher than that of ALK-5 in matured brain ECs in mice [9]. Akt knockdown has been shown to attenuate ALK-1 expression and enhance ALK-5 expression levels [91]. ALK-5 signaling in ECs also induces the expression of S1PR-1, N-cadherin and PDGF-β at certain developmental stages to build the stable vasculature [95], but it might be dispensable for the maturation of the TJs. Inhibition of TGF-β signaling in matured ECs in vivo may promote barrier maturation and prevent EndMT [90, 96].

### JAM-A-mediated *CLDN5* regulation and cAMP signaling

Trans-interacting JAM-A can induce an increase in intracellular cAMP levels, which activates CCAAT/enhancer-binding protein-α (C/EBP-α) via a regulatory factor named exchange protein directly activated by cAMP (EPAC) [52, 97]. Increased cAMP levels also activate protein kinase A (PKA), which in turn activates Rac1 (PKA-dependent TJ stabilization) [98] (Fig. 3b). C/EBP-α can enhance *CLDN5* promoter activity (PKA-independent TJ stabilization) [52]. The expression level of CLDN-5 in the brains of *Jama*^*−/−*^ mice was reported to be almost 30% of wild-type mice without VE-cadherin abnormalities [52]. EPAC-1 is a GEF for Rap1, which interacts with β-catenin and stabilizes the trans-interaction of VE-cadherin until AJ maturation is complete [97, 99]. The role of JAM-B and JAM-C for the integrity of the TJs in the BBB is still unclear: no EC-derived BBB impairment was observed in deficient mice [100, 101] but their mutations in humans clearly induce BBB impairment [102, 103].

Adenylyl cyclase, which is activated or inhibited by Gα_s_ or Gα_i/o_ coupled GPCRs, respectively, is the major source of cAMP. One representative GPCR activating Gα_s_ subunit in brain ECs is adrenomedullin receptor (*CALCRL*). Adrenomedullin is secreted by ECs and the pericytes to a lesser degree and acts as a barrier-forming molecule by elevating cAMP levels via its receptor on ECs [104]. Activated astrocytes release ATP and its hydrolysis product, adenosine, activates adenosine A2A receptor that activates Gα_s_ subunit in brain ECs [105]. Piezo-1 also induces ATP release from ECs [106]. Under acute inflammatory conditions, GPCR sensing prostaglandins were shown to be up-regulated in ECs in mice and activation by secreted or administered prostaglandins was protective following ischemia/reperfusion injury via cAMP up-regulation [107]. Phosphodiesterases (PDEs), especially PDE4 in ECs, hydrolyzes cAMP. Among them, PDE4D is localized with β-catenin/EPAC-1 complex at junctions and controls the junctional cAMP level [99].

The effect of cAMP signaling on barrier maturation is promising but there are some conflicting data in the literature [108]. Both cAMP generation and degradation by adenyl cyclase and PDEs, respectively, occur near the plasma membrane; however, there are soluble adenyl cyclase such as *ADCY10* that cause the disruption of pulmonary endothelial barrier by a currently unknown mechanism [109]. One possible mechanism is suggested in a study using prolonged exposure of cAMP up-regulators that may cause the accumulation of cAMP in the cytosolic compartment [108]; cAMP response element binding (CREB3), that is activated by abundant cytosolic cAMP, binds to the *RRAS* promoter and suppresses the expression level of R-Ras that stabilizes the trans-interaction of VE-cadherin by preventing the phosphorylation of VE-cadherin [110].

### ZO-1 and contractile/relaxation responses with RhoA, Ca^2+^ and nitric oxide signal

ZO-1 is a critical scaffolding protein for CLDN-5. After the establishment of early AJs, recruited ZO-1 and -2 make liquid-liquid phase-separated scaffolds via self-oligomerizations and associations with other junctional proteins [17]. ZOs are connected to the F-actin cytoskeleton and the rearrangement of the actin cytoskeleton by regulating myosin light chain (MLC) phosphorylation controls paracellular permeability. RhoA activates ROCKs, which phosphorylate MLC and myosin light chain phosphatase (MLCP) to deactivate it, thereby inducing cell constriction by actin-reorganization (Fig. 3c). The actomyosin-based contractile response disturbs the cortical actin belts, that is developed by Rac1 activation, generating stress fibers and redistributing the junctional proteins away from cell–cell contacts. Some GPCRs coupling Gα_12/13_ subunits, which activate RhoA, are up-regulated/activated by inflammatory mediators and neutrophils [111–113]. Calmodulin kinase, which is activated by increased intracellular Ca^2+^ levels, activates myosin light chain kinase (MLCK) to increase phosphorylated MLC levels. The release of Ca^2+^ from its store in the endoplasmic reticulum (ER) is induced by the activation of GPCRs coupling Gα_q/11_ subunits to transiently increase intracellular Ca^2+^ levels. The influx of Ca^2+^ is induced by some ion channels such as Piezo-1, transient receptor potential vanilloid 4 (TRPV4) and *N*-methyl-_D_-aspartate (NMDA) receptor. Interestingly, Piezo-1 and TRPV4 are activated by mechanical forces such as shear stress and the tension of the plasma membrane; Piezo-1 acts as an upstream regulator for TRPV4 and induces a transient Ca^2+^ influx while TRPV4 induces a sustained Ca^2+^ influx and activates associated RhoA [114, 115]. Activation of TRPV4 is inhibited by phosphatidylinositol 4,5-bisphosphate (PIP_2_) but the activation of Gα_q/11_ subunits convert PIP_2_ to inositol trisphosphate (IP_3_) [116, 117].

RhoA activation is spatially controlled by ZO-1. RhoA activators, p114RhoGEF and GEF-H1, are localized with ZO-1 via binding to cingulin and paracingulin in ECs [21, 118]. The expression level of paracingulin is much higher than that of cingulin in brain ECs [9]. Dissociation of paracingulin from ZO-1 by loss of VE-cadherin or JAM-A induced p114RhoGEF-mediated non-junctional RhoA activation in ECs [21]. Therefore, VEGF or other factors that destabilize VE-cadherin and/or JAM-A trans-interaction have the potential to activate RhoA. Of note, GEF-H1 can activate RhoA in non-junctional sites but cannot activate it at junctional sites [119]. In effect, both cingulin and paracingulin limit non-junctional RhoA activation to establish the mature barrier. GEF-H1 also interacts with ZO-1-associated nucleic acid binding protein (ZONAB) to activate it by RhoA in a ROCK-independent manner, and then promotes the translocation of ZONAB to the nucleus [120]. ZONAB binds to the *CLDN5* promoter and acts as a transcriptional repressor [121]. Well-stabilized ZO-1 interacts with ZONAB and prevents its translocation to the nucleus [17, 120]. The steady-state junctional RhoA activation and ROCKs are important for proper junction structure because the transiently localized RhoA activation is required to reanneal the TJ strands via local actin reorganization soon after their disruption by a local decrease in ZO-1 [122]. The local influx of Ca^2+^ via Piezo-1 and/or TRPV4 precedes local RhoA activation [123]. However, additional junctional RhoA activation induces peri-junctional actomyosin contraction.

PKA and cGMP-dependent protein kinase (PKG) counteracts RhoA-mediated pathogenic events by inhibiting MLCK and RhoA via phosphorylation [124, 125]. Nitric oxide (NO), which is the most important vasodilator derived from ECs to maintain appropriate CBF, activates soluble guanylyl cyclase, and then activates PKG. Both PKA and PKG phosphorylate S188 of RhoA to inhibit RhoA activation. NO is produced by endothelial nitric oxide synthase (eNOS) in ECs. The enzymatic activity of eNOS is controlled by its phosphorylation status. Akt and calmodulin kinase phosphorylate S1177 of eNOS to activate it [126, 127] and PKA phosphorylates S633 of eNOS to up-regulate its enzymatic activity [128]. The activation of TRPV4 due to the activation of GPCRs coupling Gα_q/11_ subunits depolarizes the electrical potential of the plasma membrane and inhibits plasma membrane hyperpolarization by inward rectifier K^+^ (Kir2.1) channel in ECs [116, 117]. The Kir2.1 channel can be activated by increased extracellular K^+^ ions, which is a by-product of neural activity, and the activation of ATP sensitive K^+^ (K_ATP_) channels by PKA helps the activation of Kir2.1 channels [105]. Kir2.1 can activate eNOS via PI3K/Akt signaling [126, 129]. Since the hyperpolarized electrical membrane potentials are transduced to adjacent ECs via gap-junctions, Kir2.1 activation at the BBB (capillary) induces eNOS activation in the precapillary arterial ECs and induces vasodilation via smooth muscle cells or ensheathing pericytes by NO. ROCKs phosphorylate T495 of eNOS to inhibit its activity [130]. Interestingly, RhoA activation is inhibited by eNOS-mediated NO production and vice versa. Of note, the eNOS expression level in ECs in the basal ganglia was shown to be lower than other brain regions in mice and vasoconstriction occurred only in the basal ganglia region in response to acidic blood flow mediated stimulation of the GPCR coupling Gα_q/11_ subunits in mice [131]. Therefore, attenuated eNOS levels or eNOS enzymatic activity may induce vasoconstriction instead of vasodilation by Ca^2+^-mediated signaling in ECs and cause hypoperfusion that causes angiogenic and inflammatory responses via RhoA activation.

In summary, RhoA signaling is induced predominantly by inflammatory mediators and induces cell contraction. PI3K/Akt, cAMP and Ca^2+^ signaling induce NO-mediated relaxation of the ECs and attenuate RhoA effects but the effect of these signals on barrier integrity is very different. PI3K/Akt signaling primarily contributes to building strong TJs in ECs in basal conditions. cAMP signaling also tightens the barrier and efficiently attenuates RhoA-mediated pathological effects via enhanced eNOS activity and direct phosphorylation of RhoA. A transient Ca^2+^-mediated signal transiently weakens the barrier, but sustained and/or robust Ca^2+^-mediated signal severely disrupts the barrier via RhoA activation [132]. The administration of ligands/agonists for GPCRs coupling Gα_q/11_ increases BBB permeability by modulating VE-cadherin and contractile forces with increased CBF in vivo [79, 86, 127, 133, 134]. Regadenoson, a molecule that was clinically tested as a BBB opener for patients with glioma [135], is an agonist for adenosine A2A receptor, but it induces both cAMP and Ca^2+^-mediated signaling probably via associated GPCRs [136, 137].

## The mutagenesis- and structure-based studies to characterize the CLDN-5 protein

### CLDN-5 is a representative barrier-forming CLDN

**Fig. 4:**
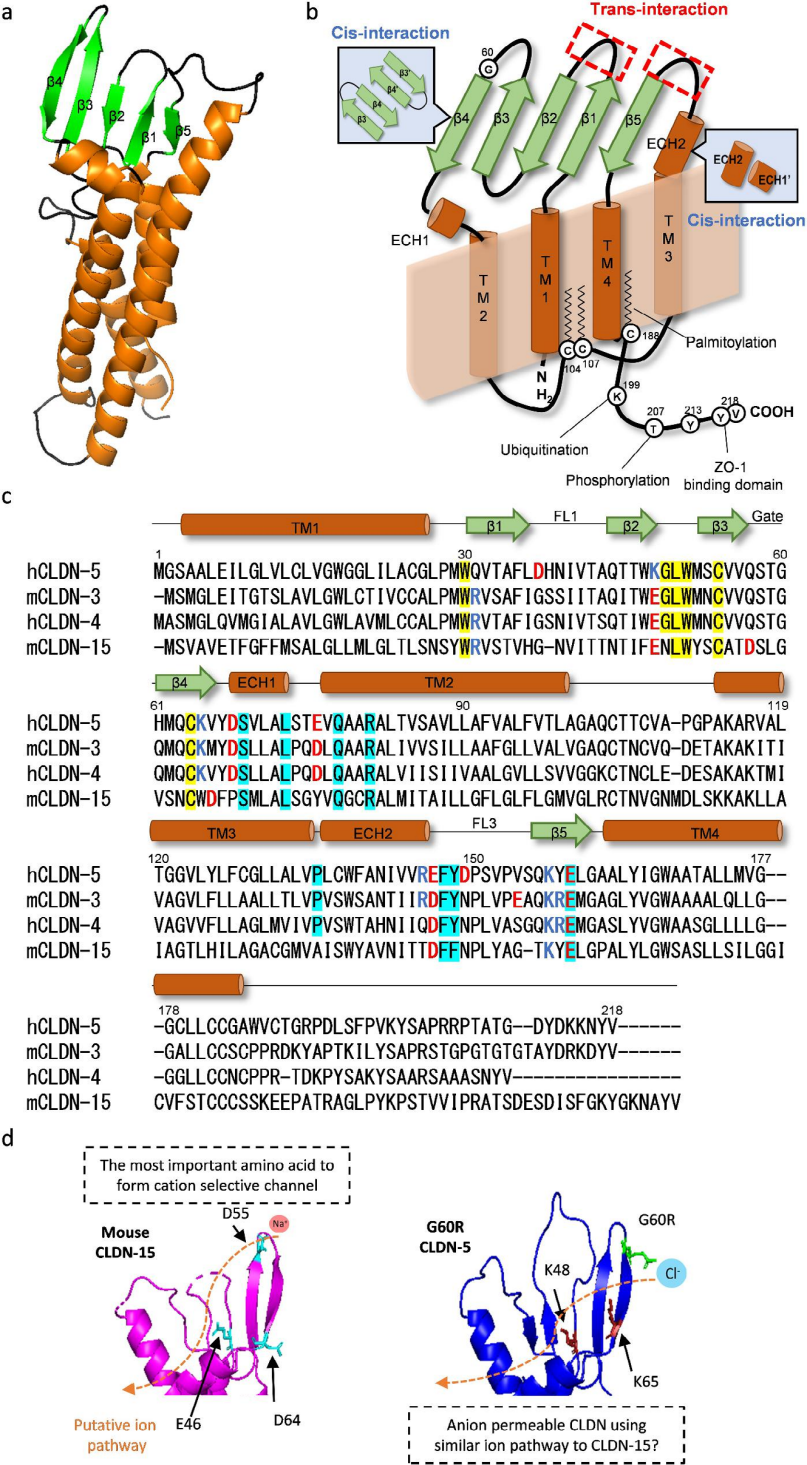
The basic information of CLDN-5 protein (a) The homology-based model of CLDN-5 based on the crystal structure of mouse CLDN-15 (PDB; 4P79) and (b) schematic illustration of CLDN-5 highlighting with key domains for forming CLDN dimers/oligomers and amino acids for post-translational modifications. (c) The sequence alignment of CLDN-5 with crystal structure confirmed CLDNs (CLDN-3, -4, and -15). The classic CLDN signature is highlighted by yellow and conserved cis-interaction sites are highlighted by cyan. (d) The similarity of a putative ion pathway between mouse CLDN-15 and CLDN-5 G60R is shown. The charged amino acids are highlighted by different colors

Since 2014, the crystal structures of some CLDNs have been described [138–142], although that of CLDN-5 is not yet known. Solving the crystal structures has dramatically improved our understanding of how CLDNs form cis- or trans-interactions. Classic CLDNs have 4 β-strands and one α-helix (extracellular helix; ECH) in their first extracellular loop (ECL) and 1 β-strand and 1 α-helix, that is like an extended transmembrane domain, in their second ECL (Fig. 4a and b). Classic CLDNs have hydrophobic W–LW residues at the beginning of the β1 strand and the tip of the β2–β3 loop: they interact at the top of the four transmembrane domains to anchor four β-sheet domains to the membrane surface. R81 in the transmembrane domain 2 is also important to maintain this interaction [143–145]. The ECH1 and ECH2 form cis-oligomers by hydrophobic interaction and the oppositely arranged two β4 strands also form cis-dimers (Table 3). An *in silico* study suggests that CLDN-5 may form different cis-interaction interfaces from other CLDNs using multiple leucine residues in the transmembrane domains 2 and 3 (leucine zipper model) [146, 147], but this model is still not yet confirmed by in vitro interaction assays using cysteine mutants or other methods [148, 149]. In mouse CLDN-15, I39 to N42 in a flexible loop between β1 and β2 strands (FL1) or F146 to K155 in a flexible loop in second ECL (FL2) forms a trans-interaction into oppositely arranged corresponding FLs [150]. The effect of mutations in the FL1 is poorly understood, but a CLDN-5 V41M mutant showed an almost comparable barrier-forming ability to wild-type CLDN-5 [151].

**Table 3:** Key amino acids for intra-, trans- or cis interaction of classic CLDNs

Amino acid positions counted by human CLDN-5 sequence	Key features	Ref
W30 and G48/L49/W50 and R81	The canonical CLDN signature to create its first ECL structure.	[138]
S69/Q78/R81 and E159	Cis-interaction by a stable hydrogen bond between S69 and E159.	[140, 150]
F139	It interacts with W30 and stabilizes the secondary structure. Only CLDN-5 has F at this position among classic CLDNs.	[146]
L73 and F147/Y148	Cis-interaction. F147/Y148 creates a hydrophobic pocket.	[140]
H61 to K65	The interface of cis-interaction for dimerization.	[229]
P135	It makes TJ strands more rigid by reducing the conformational flexibility of cis-/trans-interaction	[141]
F35, N39 to Q44	The interface of trans-interaction. The interaction of these hydrophobic residues may create a barrier against water molecules.	[148, 150]
F147 to Q156	The interface of trans-interaction. F147A, Y148A or Q156E mutant does not form trans-interaction.	[230, 231]
K65 and D68	The salt bridge (or hydrogen bond) between these positions limits the ion permeability.	[146, 232]
Q57	A polar but uncharged amino acid like Q and H is necessary for some CLDNs to form cis-interaction and to localize at the TJs.	[161, 162]
Q57 and H61 and Q63	Putative ion gate for extracellular ions is located here in channel-forming CLDNs. Barrier forming CLDNs have Q57 and Q61 (only CLDN-5 has H here) and Q63 and make multiple hydrogen bonds to close this gate.	[148]

Newly synthesized CLDNs are not directly recruited to the TJs; they are recruited to the basolateral membranes in epithelial cells [7], and then, they are incorporated into the TJs through cis- and trans-interaction/oligomerizations [148]. CLDN-5 is likely recruited to the luminal surface of brain ECs in vivo before it is incorporated into the TJs because an intravenously injected anti-CLDN-5 monoclonal antibody could disturb BBB integrity in cynomolgus monkeys, likely without a direct interaction with the TJ [152, 153]. The protein-based binding molecules against CLDNs cannot directly bind to CLDNs in the TJs due to their large size but can attenuate TJs by removing newly synthesized, monomer or dimer-form CLDNs [154]. CLDN-5 forms stronger homophilic cis-interaction compared to CLDN-1, -3 and -12 and is more strongly enriched in the TJs [149, 155]. Amino acids F139 and I142 of CLDN-5 are responsible for enhancing their strong enrichment into the TJs [149]. F139 stabilizes the secondary structure of CLDN-5 and I142 enhances cis-dimerization [146, 149]. CLDN-5 can be palmitoylated at C188 [156, 157], which can promote efficient localization into TJs [157]. The ubiquitination status of K199 also determines the destiny of internalized CLDN-5, whether it is recycled to the plasma membrane or transported to proteasomes [55].

A loop structure created by a disulfide bond between β3 and β4 strands is located at the external surface of oligomeric CLDNs and interacts with extracellular ions as a gate [147, 148, 150]. Disrupting the disulfide bond by C54S and/or C64S mutations in CLDN-5 did not induce mis-localization but attenuated the barrier against ions and solutes due to the lack of this gate structure [151]. The charged amino acids in the gate may attract ions and creates a paracellular ion channel by passing ions through junctional intramembrane particles. For example, the cation channel-forming TJ protein, CLDN-15, has a negatively charged D in the gate and the anion channel-forming CLDN-8 has a positively charged R in the gate. Barrier-forming CLDNs have three polar, but non-charged amino acids like Q or H in the gate to form multiple uncharged hydrogen bonds, probably functioning to block the interaction with extracellular ions and charged amino acids in the ECL1 of CLDNs [147, 148]. The mechanism is not yet uncovered but barrier-forming CLDNs and channel-forming CLDNs are not well intermixed in the TJ strands in the ECL domain dependent manner [4]. This may create a robust and rapid ion transport route in the TJ strands by preventing non-organized ion diffusion. It may be especially important for the peripheral ECs where CLDN-15 is also expressed with CLDN-5 [158].

### Pathogenic missense mutation of CLDN-5 and hemiplegia

Very recently, a pathogenic *de novo* mutation of *CLDN5* was originally identified by our group in two independent patients with alternating hemiplegia of childhood (AHC) [159]. The mutation in *CLDN5* (c.178G > A) is located in the gate and produces a G60R mutant. The same mutation at the same position was subsequently also found by another group that discovered 10 missense mutations and 1 frame-shift mutation of CLDN-5 (Table 4) [160]. In total, 5 patients who have a CLDN-5 G60 mutant were discovered; 4 of them developed hemiplegia and 2 of them are AHC (1 patient is identical in these two reports). Among these missense mutations, only CLDN-5 G60R mutant induces AHC or hemiplegia. The ability of CLDN-5 G60R to form a barrier against small-molecular-weight molecules (377 Da) was much weaker than that of CLDN-5 wild-type, but not completely impaired. Of note, electrical barriers created by CLDN-5 G60R had an anion preference in vitro, suggesting that patients with the CLDN-5 G60R mutation have a highly anion permeable BBB, in effect a blood-brain anion channel as opposed to a tight BBB. In CLDN-15, D55 in the gate determines the ion preference of CLDN-15; CLDN-15 D55N did not show ion preference and CLDN-15 D55K showed an anion preference [150]. CLDN-15 has two negatively charged amino acids near to D55 to support its cation interaction, and surprisingly, CLDN-5 has two K near to G60 (Fig. 4c and d). The G60R mutation may turn CLDN-5 from a barrier-forming CLDN to an anion channel-forming CLDN in a similar manner to CLDN-15.

**Table 4:** The discovered missense mutations into the coding sequence of *CLDN5* gene

Mutant	Number	Note	Ref
G60R (c.178G > A)	4	All of them developed hemiplegia and 2 of them are diagnosed with AHC.	[159, 160]
G60R (c.178G > C)	1	Not developed hemiplegia so far.	[160]
V41M (c.121G > A)	3	Its barrier forming ability was normal in epithelial cells overexpressing this mutant.	[160] [151]
Q63K (c.187 C > A)	1	The most severe phenotype was observed.	[160]
N39S (c.116 A > G)	2	It is located in the interface of trans-interaction	[160]
F35L (c.105 C > G)	1	It is located in the interface of trans-interaction	[160]
I40V (c.118 A > G)	1	It is located in the interface of trans-interaction	[160]
A43P (c.127G > C)	1	It is located in the interface of trans-interaction	[160]
V55A (c.164T > C)	1	It is located in the gate region	[160]
S58R (c.174 C > G)	1	It is located in the gate region	[160]

All patients develop epilepsy, calcium deposition in the basal ganglia and microcephaly

According to reports about the identification of pathogenic missense mutations in other classic CLDNs (CLDN-1–10, -14, -15, -17 and -19) in human, no toxic gain-of-function CLDN mutant has been reported except for CLDN-5 G60R (Table 5). Many of them are mutations into highly conserved amino acids among classic CLDNs, indicating that their loss-of-function mechanisms are also shared among classic CLDNs. CLDN-19 Q57E, which gains a charged amino acid into the gate like CLDN-5 G60R, located in the apical membrane with diffusion pattern [161]. CLDN-5 Q57D also showed severe impairment to localize at cell-cell contact [162]. The CLDN-5 Q63K mutant may also show anion channel function, but it may induce symptoms too severe for survival [160]. Therefore, converting the gate except for Q57 to the charged amino acids in CLDN-5 may become an inducer of AHC and AHC-like symptoms.

**Table 5:** The reported pathogenic missense mutations in other classic CLDNs

Identified mutation	Position in CLDN-5	Homology level	Estimated or validated changes	Ref.
CLDN-1 (R81H)	R81	Very High	Mis-localization due to the structural instability	[144]
CLDN-2 (G161R)	G161	Very high	*In silico* docking study indicates it may affect oligomer formations	[233]
CLDN-9 (E159K)	E159	Very High	Incorporation into the TJs or barrier-forming ability were not impaired. But this position needs for cis-interaction	[234]
CLDN-10a (R78G)	R81	Very High	Mis-localization due to the structural instability	[143]
CLDN-10b (N48K)	G48	High	TJs were not formed correctly due to the disturbance of classic CLDN signature	[235]
CLDN-10b (D73N)	S74	Low	Incorporation into the TJs was partially impaired by attenuation of CLDN-10b specific intra-molecule interaction	[148, 236]
CLDN-10b (P149R)	P150	Very High	Incorporation into the TJs was partially impaired by impaired cis-oligomerization	
CLDN-10b (S131L)	A132	Low	Mis-localization	[237]
CLDN-10b (G165A)	G167	Very high	Incorporation into the TJs was partially impaired and trans-interaction ability was clearly attenuated	[238]
CLDN-14 (V85D)	V85	High	Mis-localization	[239]
CLDN-14 (G101R)	G101	Very high	It localized at the cell border, but TJs were not formed correctly	
CLDN-14 (R81H)	R81	Very High	Mis-localization due to the structural instability	[145]
CLDN-19 (Q57E)	Q57	High	Mis-localization	[161]
CLDN-19 (G20D)	G20	High	Mis-localization	
CLDN-19 (L90P)	L90	High	Incorporation into the TJs was not impaired but its function was partially impaired.	
CLDN-19 (G123R)	G122	Very high	Incorporation into the TJs was not impaired but its function was partially impaired.	

AHC is a severe neurological disorder with infantile-onset (before 1.5 years of age) recurrent episodes of hemiplegia on either side of the body with episodes alternating from one side to the other. Its annual incidence is approximately 1/1,000,000 newborns [163]. *CLDN5* missense mutation is the second gene mutation to induce AHC. Importantly, the only other known factor to cause AHC is mutations into *ATP1A3*, Na^+^-K^+^-ATPase pump [164, 165], and approximately 70–80% of patients with AHC have *ATP1A3* mutations [166]. Approximately 40% of discovered AHC inducing *ATP1A3* mutations is ATP1A3 D801N mutant. The mutations into some other genes for ion transport, *ATP1A2* (Na^+^-K^+^-ATPase pump) [167], *SCN1A* (voltage-gated Na^+^ channel) [168], and *CACNA1A* (voltage-gated Ca^2+^ channel) [169] are also known to cause sporadic or familial hemiplegic migraine (HM) with symptoms very similar to AHC but with an age of onset of 2–15 years. These mutations attenuate excitability of neurons [170]. Of note, the CBF and the BBB permeability of gadolinium reagents is increased in the hemiplegic pain side of the brain in HM patients [171]. CLDN-5 G60R mutation is clearly a novel mechanism to induce AHC and it may be categorized to another sub-group of AHC. The strength of anion preference of mutated CLDN-5 may determine severity and age of onset of AHC or HM. Other undiscovered *CLDN5* mutations that may cause haploinsufficiency by in-frame deletion, miss-localization, or completely impaired barrier forming function like the mutants listed in Table 5 may not induce AHC or HM because patients with 22q11DS do not show hemiplegia. This idea is supported by the recent finding (Table 4) [160]. It is very similar to *ATP1A3* mutations-mediated AHC because missense mutations into only some specific regions of ATP1A3 cause AHC and the other mutations cause different neurological diseases with similar symptoms except for hemiplegia [172].

Ion transport by the BBB is mainly transcellular, not paracellular [173] due to CLDN-5 forming a high electrical resistance barrier. Due to these ion transporters, the cerebrospinal fluid (CSF) and brain interstitial fluid (ISF) have a higher Na^+^ and Cl^−^ concentration, a lower K^+^, Ca^2+^ concentration and equivalent HCO_3_^−^ concentration compared to plasma [174, 175]. An anion permeable BBB may efflux transported Cl^−^ and HCO_3_^−^ to blood via the paracellular route and disturb ion homeostasis by disturbing the functions of Cl^−^ or HCO_3_^−^ coupled ion transporters in the brain. Although a transporter functioning as a net transporter of Cl^−^ into the brain has not yet been identified [176], a healthy BBB maintains Cl^−^ ion homeostasis.

## Human CNS diseases induced by increased BBB permeability and CLDN-5 decline

Many neurological diseases associated with severe inflammatory responses by infiltrated neutrophils or activated microglia such as multiple sclerosis, stroke and traumatic brain injury markedly reduce CLDN-5 expression level (see our previous review [177]). These diseases-mediated CLDN-5 decline can be a leading factor of cognitive decline [178, 179]. In this review, we focus on selected neurological diseases that can be initiated by CLDN-5 decline.

### The effect of knockdown/knockout of *Cldn5* in mouse models

*Cldn5*^*−/−*^ mice die within a day of birth [8], so that no neurological diseases can be assessed using the adult animal. Mice harboring a gene coding a doxycycline-inducible short hairpin RNA (shRNA) against *Cldn5* start to die 3 weeks after the initiation of protein knockdown [180]. The CLDN-5 mRNA expression level in the mice reduced to 25% before they started to develop seizures [96]. The mice also showed several learning and memory deficits and anxiety/depression-like behavior 2 to 4 weeks after the initiation of knockdown. Interestingly, mice injected with small interfering RNA (siRNA) against *Cldn5* only showed a transient BBB opening against molecules less than 800 Da [181], but *Cldn5* knockdown mice displayed a severely disrupted BBB with severe neuroinflammation and extravasation of fibrinogen (340 kDa) [96, 180], indicating that prolonged BBB opening is enough to disrupt the brain microenvironment. Endothelial specific *Cldn5*^*+/−*^ mice have 50% less CLDN-5 protein without changes in ZO-1, ZO-2, and VE-cadherin mRNA levels [96]. The mice show normal physical/behavioral activity with weak spatial memory impairment. *Cldn5*^*+/−*^ mice do not develop seizures spontaneously, but their threshold for kainic acid-evoked seizures is greatly reduced. Although several mechanisms leading to the development of seizures have been identified in animal models, these results clearly suggest that reducing CLDN-5 expression is sufficient to develop seizures. Mice harboring a tamoxifen-inducible endothelial specific *Cldn5* knockout system have been developed recently to assess the effect of CLDN-5 knockout in peripheral ECs of adult mice [158]. Surprisingly, downregulation of CLDN-5 protein, but not *Cldn5* mRNA, was limited to approximately 75% 2 weeks after the local tamoxifen treatment. This result indicates that half-lives of TJ-incorporated CLDN-5 will be very stable when the production of CLDN-5 protein is dramatically reduced. It is likely a counteracting mechanism to compensate rapid CLDN-5 decline, but the exact mechanism is still unknown.

Stereotaxic injection of neurotropic adeno-associated virus (AAV) carrying a doxycycline-inducible shRNA against *Cldn5* is a useful technique to assess the effect of brain-region specific CLDN-5 decline on mouse behavior. *Cldn5* knockdown in the hippocampus led to a significant impairment in a spatial memory task, for which hippocampal Cornu Ammonis 1 (CA1) neurons are essential [182], and in a social novelty task, for which CA2 neurons are essential [180, 183]. The 22q11DS model mice (*Df(16)A*^*+/−*^ mice), which are haploinsufficient for *Cldn5*, also showed an attenuated firing rate of CA1 and CA2 neurons [183, 184]. *Cldn5* knockdown in the medial prefrontal cortex (PFC) induced anxiety- and depression-like behaviors [180, 185]. The spatial recognition memory was impaired by *Cldn5* knockdown in either hippocampus or medial PFC with different test results [180]. *Cldn5* knockdown in the nucleus accumbens did not induce anxiety- and depression-like behaviors but lowered resilience to the social-stress induced depression by attenuating the paracellular barrier against blood-circulating interleukin-6 (21 kDa) [186].

### The phenotype of 22q11DS and SNP *rs10314*

22q11DS is a behavioral and psychiatric disorder that includes a spectrum of cognitive defects, anxiety, attention deficit disorder, and neurodevelopmental disorders. Because this region of the chromosome is very unstable, the estimated prevalence of 22q11DS is 1/2,500–4,000 newborns. The *TBX1* gene is considered as largely responsible for the clinical findings in patients with 22q11DS, including the physical malformations and psychiatric disorders, but not cognitive impairments [187, 188]. A SNP in the 3’-untranslated region of *CLDN5* (*rs10314*) is very weakly associated with the prevalence rate of schizophrenia in many races [180, 189, 190]. The frequency of this SNP is approximately 16%. CLDN-5 expression is reduced by translational suppression because rs10314 changed the polyribosome profiling without changing the mRNA expression level [180]. Of note, 15–25% of patients with 22q11DS have experienced psychiatric disorders, mainly schizophrenia, but almost half of the patients with 22q11DS with *rs10314* (CLDN-5 expression level is further declined) have experienced schizophrenia [180, 191]. Recent dynamic contrast-enhanced magnetic resonance imagining (DCE-MRI) using gadolinium contrast agents with molecular weight less than approximately 1,000 Da enables an assessment of the subtle differences of the strength of CLDN-5-based TJs in the BBB in humans and the increased BBB permeability of gadolinium reagents clearly associated with the progression and severeness of schizophrenia and bipolar diseases [192, 193]. Therefore, *CLDN5* haploinsufficiency in 22q11DS may not be responsible for directly causing psychiatric disorders, but may be responsible for mild cognitive impairment like *Cldn5*^*+/−*^ mice [96]. Added to this, a further decline of *CLDN5* expression greatly increases the risk of psychiatric disorders like *Cldn5* KD mice [180].

### Mild cognitive decline

The BBB permeability of gadolinium reagents was specifically increased in hippocampal regions, especially in the CA1 and dentate gyrus, in patients with early phases of cognitive decline [85]. Added to this, indirect evidence that the trans-interaction of VE-cadherin and the interaction of PDGF-β and PDGFR-β were attenuated was observed by measuring soluble PDGFR-β, which can be an indicator of ADAM-10 activity, in the CSF of these patients. Therefore, increased BBB permeability in hippocampal regions maybe enough to cause mild cognitive decline in humans. A recent report using a direct live imaging of hippocampal capillary in mice clearly showed that the capillary density and resting CBF in CA1 region in hippocampus were lower than that in the neocortex in mice, although the oxygen consumption in resting conditions were equivalent between them [194]. Added to this, the distance between the soma of pericytes and the length of the pericyte processes in the hippocampal BBB were significantly greater than those in the neocortex BBB in mice [194], indicating that hippocampal ECs may receive weaker PI3K/Akt signaling compared to the other brain regions. This finding may be the reason why ECs in the CA1 are especially vulnerable against pathological stimuli, such as hypoxia and decreased CBF, in the brain capillaries [195]. The mRNA expression of Kir2.1 and soluble guanylyl cyclase were lower in the hippocampal ECs in mice and resulted in weaker and less frequent NVC-mediated dilation [194]. The activity of Kir2.1 is also suppressed by cholesterol and it has been shown that hypercholesterolemia attenuates Kir2.1-induced vasodilation [196]. In effect, the attenuated Kir2.1-mediated NVC response is one of the pathogenic effects induced by the *APOE4* allele, which is a well-known risk factor for hypercholesterolemia and cognitive decline, and may injure the ECs and pericytes by chronic mild hypoperfusion [197].

It is still unresolved whether hippocampal ECs are especially vulnerable against healthy aging related processes, but increased BBB permeability in the white matter and associated loss of myelin by aging also contributes towards the onset of dementia [198, 199]. Alzheimer’s diseases (AD) is known to cause vascular-mediated dementia. DNA methylations in *CLDN5* region have been observed even in non-hippocampal regions in AD patients [200]. Aggregated amyloid-β causes CLDN-5 downregulation in ECs [201] and causes a decrease in CBF by pericyte-mediated vasoconstriction [202] or by neuroinflammation-mediated loss of pericytes [203]. Pericytes are also vulnerable against extravasated fibrinogen from a compromised BBB [204]. In mice with mutations in PDGFR-β resulting in pericyte loss in an age-dependent manner, the BBB in the white matter is more vulnerable than the BBB in the hippocampus [204]. Cerebral small vessel disease (cSVD) is the second cause of vascular-mediated dementia. It can be induced by impaired NVC-mediated chronic mild hypoperfusion by aging, and *eNos*^*−/−*^ or *eNos*^*+/−*^ mice have been used as a model of spontaneous cSVD. In these mice, severe vascular constriction is observed in the hippocampus and temporoparietal and retrosplenial cortices [205] and severe myelin loss in the white matter is also induced, consistent with the human condition [206]. The BBB permeability of a gadolinium reagent was increased and CBF was decreased in the white matter of individuals who had experienced ischemic insults within the previous 2 years of the MRI study compared to that of the control participants or patients with Parkinson disease [207]. Evidently, the BBB in the white matter is also especially vulnerable against hypoxia and the fibrous astrocytes that mainly present in the white matter are known to be more vulnerable against ischemic insults compared to protoplasmic astrocytes [77]. Contrary to this, ECs in the white matter have a higher CLDN-5 expression level than ECs in the grey matter in the same brain region in both humans and mice [78, 208]. ECs in the white matter are well-supported by other cells to express CLDN-5 in the basal conditions, but they cannot efficiently recover from pathogenic effects induced by mild hypoperfusion likely due to the difference of tolerability against hypoxic damage. RhoA signaling may then be heightened in ECs and pericyte numbers are reduced due to extravasated fibrinogen.

Ultimately, the BBB in the hippocampus is vulnerable against hypoxia due to its vascular structure and reduced pericyte coverage. The BBB in white matter is also vulnerable against hypoxia partially via loss of fibrous astrocytes. Once microbleeding occurs at the BBB, pericytes are injured and CLDN-5 expression is reduced due to impaired EC–pericyte interactions.

### Psychosis and depression

Stress causes the induction of inflammatory cytokines and neurotransmitter disturbances, resulting in reduced CLDN-5 expression. Schizophrenia, whose clinical hallmark is psychosis, is one of major psychiatric disorders globally. Further CLDN-5 decline in the hippocampal sub-region in patients with dementia may induce schizophrenia. Approximately 40–60% of AD patients have experienced psychosis and these patients showed more rapid and severe cognitive decline [209] and approximately 70% of patients with schizophrenia showed cognitive decline [210]. A study using post-mortem human brain sections showed that CLDN-5 expression level was attenuated in the grey matter in the hippocampus, but not in the cortex, in the patients with schizophrenia [208]. Of note, 22q11DS patients who have experienced schizophrenia showed severe cognitive decline before they developed psychosis compared to 22q11DS patients who have not experienced schizophrenia [191]. Thus, cognitive decline may precede the onset of schizophrenia, which is induced by further CLDN-5 decline in the hippocampus by severe hypoperfusion and other stimuli. PDE4B and PDE4D inhibitors, which upregulate CLDN-5 level by cAMP-mediated pathway, are considered as potential drugs for schizophrenia and dementia [211].

Major depressive disorders (MDD) and bipolar disorder induces depressive symptoms. MDD is also a major psychiatric disorder, but unlike schizophrenia, MDD is not clearly associated with the onset of dementia [212]. Approximately 35% of MDD patients have schizophrenia with significantly worse scores for anxiety and mood disorders [213] and more than 80% of patients with schizophrenia have experienced depression during the early phase of the disease [214]. The smaller volume of the hippocampus and CLDN-5 decline in this region was also observed in patients with MDD [208, 215], but the volume reduction of CA1, CA3, dentate gyrus and total hippocampus, but not CA2, was less severe compared to patients with schizophrenia [216]. Thus, depression may be induced by milder CLDN-5 decline compared to cognitive decline and schizophrenia. CLDN-5 mRNA and protein level were reduced in the nucleus accumbens in patients with MDD owing to *CLDN5* promoter methylation [185, 217]. This epigenetic suppression was also shown to be induced by β-catenin/FoxO1 repressor in mice with mild social defeat stress [217]. Of note, the decline in both CLDN-5 mRNA and protein was also observed in the ventromedial PFC in only female patients with MDD [185]. It may be consistent with the fact that women are more vulnerable to MDD than men [213]. A traditional mood stabilizer, lithium, functions as a mild GSK-3β inhibitor and an anti-depressant, fluoxetine, could induce GDF-15 from astrocytes [69, 218]. Both activate PI3K/Akt signaling pathway, and then inhibit RhoA-mediated cell contraction by eNOS activation.

### Epilepsy

As 10–25% of patients with 22q11DS develop epilepsy [219] and *Cldn5* KD mice develop epilepsy [180], CLDN-5 decline might be an initial trigger for seizures and epilepsy due to the extravasation of blood-borne proteins or induction of neuroinflammation [220], but it mainly contributes to increasing the severity and vulnerability of recurrent epilepsies. Epilepsy disrupts the BBB adjacent to epileptic foci via abnormally released glutamate because NMDA receptor mediated Ca^2+^ influx and RhoA activation can also occur in the ECs [96, 221]. Importantly, almost half of patients with AHC develop epilepsy, but all patients with CLDN-5 missense mutations have epilepsy [159, 160]. According to a meta-analysis, 24.2% and 1.7% of epilepsy patients have MDD and schizophrenia, respectively [222]. The distance between the hippocampus and epileptic foci is also important for the co-morbidities of these diseases; patients with temporal lobe epilepsy have a higher prevalence of psychiatric comorbidities than patients with extratemporal lobe epilepsy [223]. The TGF-β inhibitors, such as RepSox, may restore CLDN-5 expression in ECs and might inhibit abnormal TGF-β-mediated epileptic signaling in the astrocytes by extravasated proteins at the same time [96, 220].

### Brain calcifications in the basal ganglia

Calcium phosphate deposition develops around the BBB preferentially in the basal ganglia and, to a lesser extent, in the cerebellum and white matter in patients with gene mutations into the phosphate transporters/exporters or TJ proteins and their supporters [22]. The major symptoms are depression, anxiety, headache, psychosis and cognitive decline and their severities generally correlate with the number of calcified areas. It is still unknown why the BBB in the basal ganglia is highly vulnerable for brain calcification. Patients with CLDN-5 missense mutation also developed brain calcification in the basal ganglia [159, 160], but, interestingly, brain calcification in the basal ganglia is not necessarily a common hallmark observed in AHC patients with *ATP1A3* mutations or 22q11DS patients. The other gene mutations relating to BBB permeability are occludin (*OCLN*) [224], JAM-B (*JAM2*) [103], JAM-C (*JAM3*) [102], PDGF-β (*PDGFB*) [225], and PDGFR-β (*PDGFBR*) [226]. Non-genetic causes of brain calcification can be observed in the hippocampus in over 20% of people over 50 years of age [227]. It is strongly correlated with cognitive decline and its risk is increased by hypoperfusion, hypertension and hypercholesterolemia, indicating that it is likely induced by the consequences of increased BBB permeability.

## Conclusions

Enriched expression of CLDN-5 is one of the key features of the BBB, but this key feature of brain ECs is lost without the support of cells from the neurovascular unit including pericytes and astrocytes. There is heterogeneity in the extent of pericyte coverage, the tolerability against ischemic insults and neurons among the brain regions. These differences make certain BBB regions in the brain vulnerable by attenuating PI3K/Akt, cAMP and NO signaling, and subsequently initiating some pathogenic events with increased BBB permeability and RhoA activation in a brain-region specific manner. Added to this, it is clear that CLDN-5 decline exacerbates the pathology of many CNS diseases that disrupt CLDN-5 expression by cytokines or immune responses. A comprehensive understanding of CLDN-5 based TJs in the ECs and its regulation by pericytes and astrocytes should lead to novel drug targets to treat vascular-mediated dementia, schizophrenia and MDD and to reduce some of the pathologies of other CNS diseases. Mutations in CLDN-5 are clearly pathogenic in humans. It is now likely that other channel-forming CLDN-5 mutants will be discovered as a causative factor for AHC or HM or novel loss-of-function CLDN-5 mutants may be discovered as a causative factor for mild cognitive decline or brain calcification in the basal ganglia. Although it is still challenging to selectively target the vasculature in the body, gene replacement therapies using adeno-associated virus are now in clinical development with some already FDA approved [228]. Once a suitable vector is developed, CLDN-5 may be incorporated into the vector easily to allow for regulated expression of the protein and stabilization of the BBB.

## Abbreviations

22q11DS: 22q11 deletion syndrome
AAV: Adeno-associated virus
AD: Alzheimer’s disease
ADAM: A disintegrin and metalloproteinase
AHC: Alternating hemiplegia of childhood
AJ: Adherens junctions
ALK: Activin receptor-like kinase
BBB: Blood–brain barrier
BMAL1: Brain and muscle aryl-hydrocarbon receptor nuclear translocator like protein 1
CA: Cornu Ammonis
CBF: Cerebral blood flow
C/EBP-α: CCAAT/enhancer-binding protein-α
CLDN: Claudin
CNS: Central nervous system
CSF: Cerebrospinal fluid
cSVD: Cerebral small vessel disease
DCE-MRI: Dynamic contrast-enhanced magnetic resonance imagining
EC: Endothelial cell
ECH: Extracellular helix
ECL: Extracellular loop
EndMT: Endothelial-to-mesenchymal transition
eNOS: Endothelial nitric oxide synthase
EPAC: Exchange protein directly activated by cAMP
ERG: E-26 transformation specific related gene
ETS: E-26 transformation specific
GDF-15: Growth/differentiation factor-15
GEF: Guanine nucleotide exchange factor
GPCR: G-protein coupled receptor
GSK-3β: Glycogen synthase kinase-3β
HM: Hemiplegic migraine
IP_3_: Inositol trisphosphate
JAM: Junctional adhesion molecule
JNK: c-Jun N-terminal kinases
KLF-4: Krüppel-Like Factor 4
LRP: Low-density lipoprotein receptor-related protein
MDD: Major depressive disorder
MLC: Myosin light chain
MLCK: Myosin light chain kinase
MLCP: Myosin light chain phosphatase
MMP: Matrix metalloprotease
NF-κB: Nuclear factor κB
NMDA: *N*-methyl-_D_-aspartate
NO: Nitric oxide
NVC: Neurovascular coupling
PDE: Phosphodiesterase
PDGF-β: Platelet-derived growth factor-β
PDGFR-β: Platelet-derived growth factor receptor-β
PFC: Prefrontal cortex
PI3K: Phosphoinositide 3-kinase
PIP_2_: Phosphatidylinositol 4,5-bisphosphate
PKA: Protein kinase A
PKG: cGMP-dependent protein kinase
ROCK: Rho-associated kinase
RUNX1: Runt-related transcription factors 1
S1P: Sphingosine-1-phosphate
S1PR1: Sphingosine-1-phosphate receptor 1
shRNA: Short hairpin RNA
siRNA: Small interfering RNA
SNP: Single nucleotide polymorphism
SOX-1: Sex-determining region Y-box 18
TGF-β: Transforming growth factor-β
TGFBR-2: Type II transforming growth factor-β receptor
TJ: Tight junction
TRPV4: Transient receptor potential vanilloid 4
VEGF: Vascular endothelial growth factor
VEGFR2: Vascular endothelial growth factor receptor 2
Wnt: Wingless/int-1
ZO: Zonula occludens
ZONAB: Zonula occludens-1-associated nucleic acid binding protein
